# A Novel Scoring System for the Administration of an IgM- and IgA-Enriched Intravenous Immunoglobulin Preparation: The SORRISO Score

**DOI:** 10.3390/jcm14175950

**Published:** 2025-08-22

**Authors:** Mattia Bixio, Lucio Torelli, Alice Scamperle, Giada Quarantotto, Silvia Zanchi, Silvia Baronio, Lucia Mirabella, Alessandro Conti, Francesco Forfori, Alberto Noto, Valeria Bonato, Andrea Cortegiani, Eugenia Botter, Antonino Chillemi, Irene Longo, Roberto Dattola, Giorgio Berlot

**Affiliations:** 1 UO Anestesia e Rianimazione, Ospedale Policlinico San Martino, 16132 Genova, Italy; mattia.bixio@hsanmartino.it; 2 Dipartimento Universitario di Scienze Mediche, Chirurgiche e della Salute, Università di Trieste, 34129 Trieste, Italy; torelli@units.it; 3 Department of Anesthesia, Intensive Care and Pain Therapy, Azienda Sanitaria Universitaria Giuliano Isontina, 34148 Trieste, Italy; alicescamperle@libero.it (A.S.); zanchi.sil1@gmail.com (S.Z.); baronio.silvia92@gmail.com (S.B.); eugeniabotter@gmail.com (E.B.); irenelongo.ing@gmail.com (I.L.); robdattola@gmail.com (R.D.); 4 Department of Anesthesia and Intensive Care, Pordenone Hospital, 33170 Pordenone, Italy; giadagg48@gmail.com; 5 Anesthesia and Intensive Care, Department of Medical and Surgical Science, University of Foggia, 71121 Foggia, Italy; lucia.mirabella@unifg.it; 6 UO di Rianimazione, Ospedale San Marco, 95121 Catania, Italy; alecontict@hotmail.com; 7 Dipartimento di Patologia Chirurgica Medica Molecolare e dell’Area Critica, Università di Pisa, Azienda Ospedaliero Universitaria Pisana, 56126 Pisa, Italy; francescoforfori@gmail.com; 8 Division of Anesthesia and Critical Care, Department of Human Pathology of the Adult and Evolutive Age “Gaetano Barresi”, University of Messina, 98122 Messina, Italy; alberto.noto@unime.it; 9 SS Terapia Intensiva Polivalente Anestesia e Rianimazione, Azienda Ospedaliera Universitaria SS Antonio e Biagio e Cesare Arrigo, 15121 Alessandria, Italy; v.bonato@ospedale.al.it; 10 Department of Precision Medicine in Medical, Surgical and Critical Care Area (Me.Pre.C.C.), University of Palermo, 90127 Palermo, Italy; andrea.cortegiani@unipa.it; 11 General Intensive Care Unit, Policlinico Paolo Giaccone, 90127 Palermo, Italy

**Keywords:** septic shock, intravenous immunoglobulins enriched with IgA and IgM (eIg), SORRISO Score, patient stratification, early treatment, antibiotic adequacy, immunosuppression

## Abstract

**Background/Objectives**: The use of intravenous immunoglobulins enriched with IgA and IgM (eIg) in patients with septic shock remains controversial due to a lack of robust, standardized criteria for patient selection and the timing of treatment. This study introduces the SORRISO Score, which is a novel, evidence-informed scoring system designed to guide clinical decision-making for the administration of eIg. **Methods**: Based on data from the Italian multicentric SORRISO registry, involving 248 patients across seven ICUs from 2015 to 2022, the score integrates patient-related and therapy-related variables. These were derived through an enhanced version of the TO-PIRO Score and include factors such as immunosuppressive status, infection type, timing of treatment, and adequacy of antibiotic therapy. **Results**: Statistical analyses, including Kaplan–Meier curves and regression models, identified the key predictors of survival and validated the score’s ability to stratify patients by outcome. A cutoff of 13.7 showed a significant prognostic value (AUC = 0.731), with lower scores correlating with increased mortality. **Conclusions**: The SORRISO Score thus offers a practical bedside tool for improving patient selection for eIg therapy, potentially optimizing outcomes in septic shock; however, it should be validated in a larger cohort of patients.

## 1. Introduction

The guidelines of the Surviving Sepsis Campaign (SSC) discourage the administration of intravenous immunoglobulins (IvIgs) in patients with sepsis and septic shock primarily because the studies published in the literature do not fulfill the evidence-based criteria (EBM) [1]. Many randomized clinical trials (RCTs) are biased on a number of counts [2], including the heterogeneity of the patients enrolled, the different compositions of IvIgs used, the variable timing of their administration, the sources of sepsis, and the pathogens involved, among others. The current definition of sepsis and septic shock considers these clinical entities to be the consequence of a derangement of the immune system; the definition is wide enough to cover the hyperinflammatory phase and the immunosuppression that often appears in the advanced states of intensive care unit (ICU) admission [3]. Consequently, IvIg could be valuable for both biological and clinical reasons. The biological reasons include their antibacterial and opsonizing capabilities and immune-modulating activities, which make them suitable in both phases of sepsis and during the immunoparalysis that often follows the initial insult [4]. The clinical reasons are based on a number of investigations demonstrating that (a) low values of endogenous Ig are associated with a worse outcome in septic patients [5]; (b) their decrease and/or failed increase in response to an infection is associated with a shift from sepsis to septic shock [6,7]; (c) septic patients administered IvIg have a better outcome compared with untreated patients [8]; (d) this survival advantage is more marked in patients treated with a preparation enriched with higher-than-normal amounts of IgA and IgM (eIg) [9,10]; and, finally, (e) early treated patients have a better outcome compared with those administered eIg in a more advanced phase of their clinical course [11]. A major matter of concern for the use of this expensive preparation consists of the absence of precise indications for their initiation, making the decision to start their administration somewhat arbitrary. As a consequence, this approach can lead to the administration of eIg to moribund patients or, conversely, to their use being withheld in patients who may benefit from the proteins in a wide array of clinical settings in which eIg could represent a valuable adjunctive therapy [12].

To fill this research gap and provide some rules of engagement (ROEs) that can be easily applied at the bedside, we used the data obtained from a large number of patients who were administered eIg in a multi-center observational study.

## 2. Materials and Methods

This study consisted of three phases. In the first phase, we recorded the clinical course, number of biological variables, and the outcome of sepsis and septic shock patients administered eIg who were enrolled in the Italian Multicentric SORRISO (Studio Osservazionale Registro Rianimazioni Italiane Sepsi Ospedaliere) observational registry. This was performed in Italy from May 2015 to March 2022 and involved 7 ICUs; the aim was to study the effects of administering eIg in patients with septic shock (Pentaglobin^®^, Biotest, Dreiech, Germany). All data were gathered through the REDCap (Research Electronic Data Capture) platform, a secure web application designed for data collection and management in research studies.

The evaluation of eIg therapy was based on a structured and time-sensitive data collection process using the REDCap platform. Case Report Forms were designed to capture key clinical parameters at specific time points throughout the patient’s hospitalization and treatment. These included baseline data at enrollment, detailed sepsis assessments at hospital and ICU admission, and daily monitoring forms tailored to both pre-treatment and post-treatment phases. In-depth immunological evaluations, including lymphocyte population analysis and immunoglobulin levels, were scheduled on critical days (e.g., days 0, 4, 7, 14), while simplified forms are used on intermediate days to ensure continuous tracking of clinical progress.

Each patient admitted to the Intensive Care Units (ICU) of the participating centers with septic shock or severe sepsis was considered eligible for the administration of eIg. The exclusion criteria were as follows: aged < 18 years; being pregnant; having known reactions to blood and its derivates; and having a life expectancy < 3 months.

The severity of the patient’s clinical condition at admission was assessed using the SAPSII Score. The diagnosis of septic shock was based on the current definitions [3]. The diagnosis of sepsis-induced coagulopathy (SIC) was based on a platelet count < 100,000/mL and a spontaneous INR > 1.5 [13].

Microbiological isolations were systematically recorded, along with detailed antibiotic therapy data, including timing, dosage, and appropriateness based on culture results. This comprehensive and temporally structured approach enabled a dynamic analysis of both immunological and infectious parameters throughout the patient’s clinical course.

The antibiotic therapy was defined as adequate when (a) it was active against at least 75% of the culture isolates or (b) was coherent with the current guidelines for a determined infection. Multidrug resistance (MDR) was defined as acquiring non-susceptibility to at least one agent in ≥3 antibiotic categories [14].

**Figure 1:**
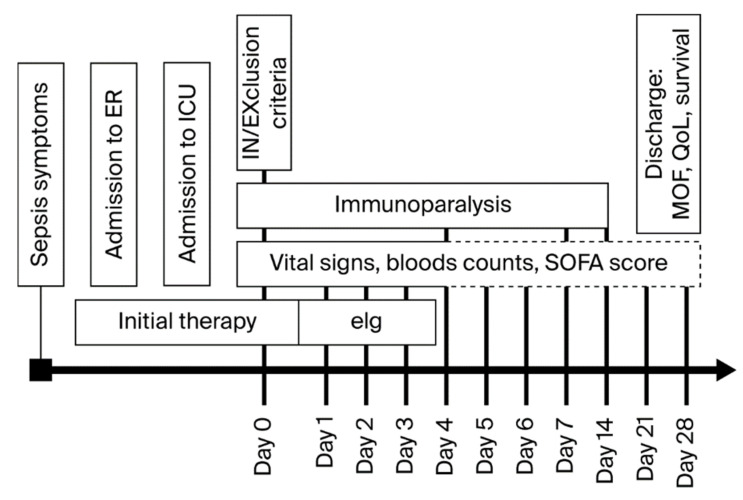
Timeline of the SORRISO study.

The decision to administer eIg was left to the attending staff, but since the start of the first infusion, all ICUs have followed the same monitoring protocol (Figure 1).

Apart from the administration of eIg, each ICU was allowed to follow its own internal procedures, including the use of blood purification (BP) techniques with the exclusion of therapeutic plasma exchange as this procedure removes all plasma content.

eIg were administered at a fixed dose of 250 mg/kg for 3 consecutive days; the duration of each infusion varied from 12 to 24 h. As eIg are not removed or absorbed, their administration was uninterrupted during the BP procedure. The patients were subdivided into early and late starters (ES and LS) using a cutoff value of 72 h from their admission to the hospital.

In the second phase of this study, we introduced some therapy-centered variables that were recorded in the SORRISO database and significantly different between survivors (S) and non-survivors (NS), including the timing of the eIg initiation and the adequacy of the antibiotic therapy, to a pre-existing score (TO-PIRO) [15]. The TO-PIRO Score represents a modification of the PIRO system of the description of the underlying conditions of septic patients [16]. It considers four different items (Predisposition, Insult, Response, and Organ failure) that are evaluated for their influence on the patient’s outcome. In the TO-PIRO Score, developed with an experts’ panel, each variable is given a score, the sum of which indicates whether there is an opportunity to administer eIg within a determined time window.

In the last phase of this study, all the statistically significant or near-significant variables demonstrated in this revised TO-PIRO Score (rTO-PIRO) were analyzed and used to build up the SORRISO Score.

Statistical analyses were performed using Jamovi software version 2.3.28, unless otherwise indicated.

## 3. Results

In total, 248 patients were enrolled in this study (Table 1).

The most significant differences among the different centers include the age of the patients (*p* = 0.009), the sites of infection (*p* < 0.001), and the proportion of ICU admissions for medical or surgical pathology (*p* = 0.058), with a borderline significant value, whereas the severity of the cases did not differ among the centers (*p* = 0.799); the lack of precise enrollment criteria likely accounted for these variations. In the overall population, the appropriateness of the antibiotic treatment was 85%.

The coordinating center in Trieste alone enrolled about two-thirds of the total number of patients. Therefore, other important criteria that could potentially affect the primary outcome (28-day hospital survival) were also considered. Among these variables, the most significant were the presence of septic shock at the onset of therapy and the precocity of the treatment (hours between hospital admission and treatment initiation), with an early vs. late cutoff of 72 h. No adverse events were reported.

The last two factors exhibited highly significant differences between the coordinating center and other hospitals (*p* < 0.001). The coordinating center admitted more septic shock patients who received eIg within a shorter treatment-free interval. These factors seem to counterbalance each other, resulting in there being no significant impact on the primary outcome at 28 days (*p* = 0.165).

The 28-day and in-hospital mortality rates were 38.3% and 44.4%, respectively.

Following this descriptive analysis, the impact of various factors on the response to eIg therapy was more comprehensively assessed using Kaplan–Meier curves for the binary parameters (presence/absence), either grouped by homogeneous clusters or based on a cutoff value.

Subsequently, the TO-PIRO Score [15] was modified by adding in some variables identified by the SORRISO Project Board (Table 2). The items included in this revised score (rTO-PIRO) included the following: (a) the presence of any kind and any stage of solid or hematologic neoplasms; (b) the presence of immunosuppression of whatever cause; (c) the blood levels of only the C-reactive protein (PCR) and procalcitonin (PCT); and (d) a simplified DIC score for the SIC evaluation [13].

Subsequently, the influence of each component of the rTO-PIRO Score on the outcome was analyzed separately.

### 3.1. Predisposition Criteria

No difference in mortality was observed among the different centers. Similarly, neither gender (*p* = 0.72), age groups (*p* = 0.12), BMI (*p* = 0.94), nor colonization with MDR pathogens or fungi (*p* = 0.75) were associated with the outcome. The presence of either solid or hematologic cancer was associated with near-significant increased mortality (*p* = 0.078). However, any kind of immunosuppression was highly correlated with the outcome (*p* = 0.00042).

### 3.2. Insult Criteria

Survival was significantly different between patients with surgical or medical ICU admission (*p* = 0.016). A near-significant better outcome was observed in patients with abdominal vs. non-abdominal infections (*p* = 0.067); this trend became significant in patients with primary or secondary peritonitis (*p* = 0.036). The involvement of a Gram-negative or MDR strain was associated with a better outcome (*p* < 0.0001 and =0.0017, respectively).

### 3.3. Response Criteria

A SAPSII Score on admission and the presence of SIC were associated with increased mortality (*p* < 0.0001). However, the baseline IgM values, PCT, and PCR were not associated with the outcome.

### 3.4. Organ Criteria

Septic shock and the blood lactate levels were not significantly different between survivors and non-survivors (*p* = 0.081 and 0.096, respectively).

### 3.5. Treatment Criteria

The 28-day survival and in-hospital survival were significantly better in ES than in LS (*p* = 0.04 and 0.025, respectively), as well as in the case of appropriate antibiotic therapy (*p* = 0.021).

### 3.6. SORRISO Score Development

The variables of the rTO-PIRO Score that were significantly or near-significantly different between S and NS in the Kaplan–Meier analysis were weighted according to the ODDS ratio within the univariate analysis to develop the SORRISO Score (see Table 3). 

The SAPSII Score was excluded because it is already an extensively validated predictor of mortality and because the score is also based on some of the parameters already included individually. It was not possible to include any organ criteria in the score.

The SORRISO Score was calculated by summing the value of individual items with a range from 0 (worst) to 20.3 (best).

**Figure 2:**
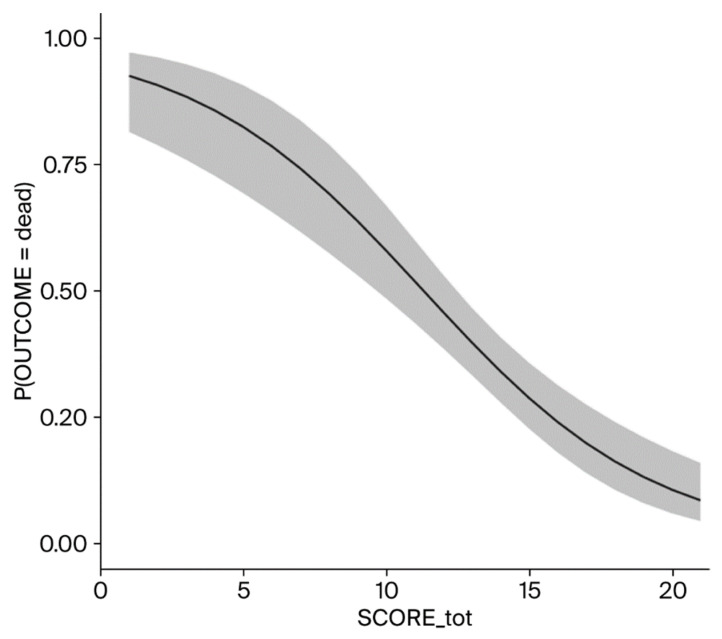
Estimated marginal means (SORRISO Score).

Binomial linear regression with a marginal mean estimation (Figure 2) was performed; it showed an excellent correlation (*p* < 0.001) between the increase in the SORRISO Score and the reduction in case fatality.

The ideal cutoff for the best sensitivity and specificity was then identified as 13.7 points, with an appreciable AUC of 0.731 at the ROC curve, using Cox Regression and Optimal Cut-Point Analysis on the Jamovi Survival module (see Appendix A).

**Figure 3:**
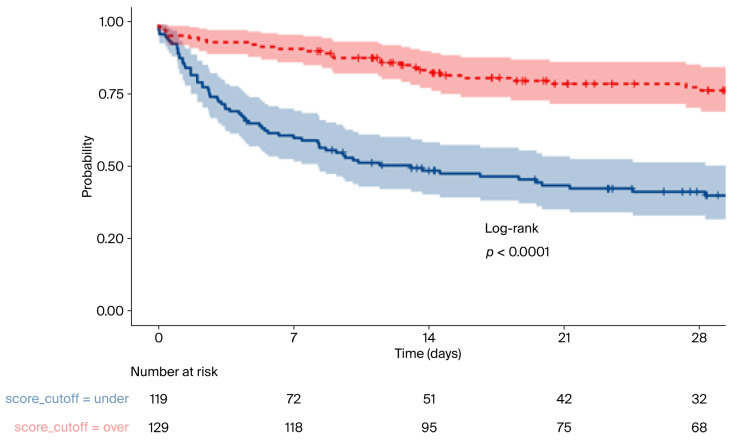
Kaplan–Meier curve for patients with scores above or below the cutoff.

To further confirm the validity of the score and its cutoff, a Kaplan–Meier curve with high significance (*p* < 0.0001) was produced (Figure 3).

Considering 28-day mortality, the lowest-scoring group had 57.1% mortality and the highest-scoring group had only 20.9% mortality, showing a clear distinction from the mean value of all patients of 38.3%.

## 4. Discussion

Despite what the SSC suggests, eIg are largely used in septic patients on the basis of a number of studies and meta-analyses that demonstrate their efficacy in different subsets of patients [8,9,10], either alone or in combination with other adjunctive treatments, including BP [17]. However, the decision as to whether eIg should be administered remains somewhat arbitrary due to a lack of precise indicators. Consequently, the use of eIg in septic shock looks to be more of an act of faith rather than a rational choice relying on sound scientific bases.

To overcome this subjective approach, the use of a number of possible triggers has been suggested that can be subdivided into four main categories, each with their own advantages and limitations.

The first category consists of the restoration of normal Ig values, as low levels have been associated with increased mortality in septic patients [7]. However, it remains unclear whether ‘normal’ Ig levels are truly sufficient in this context, or if supraphysiological levels might be more beneficial. If the latter is true, Ig administration could be justified even in patients with baseline physiological levels. The second category is based on the measurement of circulating levels of light λ chains, for which an increase indicates the failed production of mature Ig molecules [18]. Even if this approach has a sound biological rationale, it is expensive, time-consuming, and is not available on a 24H/7D basis, thus exceeding the relatively narrow window for the opportunity to administer eIg.

The third category considers some clinical circumstances in which eIg have been proven to be clinically effective both in the florid phase of sepsis and during the more advanced stage of immunoparalysis [12].

The fourth category uses a score, based on an expert’s opinion, whose final value indicates the opportunity to initiate eIg immediately or to delay the treatment [15].

We used this last approach as a starting point that was modified by adding some patient and treatment-related variables that differed between S and NS in the SORRISO study. Some of these (i.e., the treatment-free interval and the prevalence of MDR infections) present wide variations among the different centers, but these differences are valuable as they more accurately reflect the real-world scenario of a busy ICU rather than an RCT, in which the patients are enrolled and treated according to a rigid and predetermined protocol.

On the basis of these results, according to the rTO-PIRO Score, the following can be stated:

- The timing of treatment appears to be particularly relevant, as a longer eIg-free interval has been associated with a worse outcome [11]; the same also applies for the initiation of an appropriate antibiotic [19].
- Surgical patients, even when the infection is caused by MDR strains, have a better outcome compared with medical patients, especially if the site is abdominal (peritonitis) and related to Gram-negative bacteria.
- Immunosuppressed patients, independently from the underlying causes and oncologic patients, respond poorly to eIg; we do not have an explanation for this finding, but we can hypothesize that even if the humoral part of the adaptative arm of the immune system can be restored, the other components are too compromised to take advantage form the eIg.
- The same applies for patients with SIC, which is considered to be a consequence of the activation of the coagulative cascade by the septic mediators [13]. Among the different items considered in the SORRISO Score, SIC represents the only condition that can be modified by the treatment.
- The levels of IgM, C--reactive protein and Procalcitonin, as well as lactate dosage and norepinephrine requirements, do not appear to be useful trigger criteria for initiating or delaying the administration of eIg.

The subsequent development of the SORRISO Score provides a cutoff value separating patients with different outcomes and thus represents a potential indication for eIg. Given that all patients enrolled in the SORRISO study were treated according to the manufacturer’s recommendations, we believe it is important to emphasize that the presence of conditions associated with poor outcomes, as suggested by the SORRISO Score, should not automatically preclude or discourage treatment. Rather, these conditions should prompt a thoughtful use of eIg, tailored to the individual clinical context. In fact, among the different items that were used to calculate the SORRISO Score, SIC represents the only modifiable variable, meaning that it constitutes a potential indicator of the efficacy of the treatment.

One limitation of our study is that there may be an indication bias due to the lack of randomization, as the decision to administer eIg was exclusively based on the single center’s experience.

The SORRISO Score is a registry-based proposal, derived from and validated using the same cohort, and an explorative tool that must be validated using a greater number of septic patients.

## 5. Conclusions

The SORRISO Score is an exploratory tool that combines patient-related and treatment-related variables, selected for their association with outcomes that are relatively easy to assess and monitor in clinical practice, especially during the early phases of sepsis. Its structure is designed to support timely clinical decision-making and, possibly in the future, guide the appropriate use of eIg. It has been shown that it can identify subjects with different severity profiles and, consequently, different opportunities for taking advantage of the time windows in which eIg can be administrated. However, this is an exploratory study whose results must be confirmed in a larger population of patients.

## Figures and Tables

**Table 1:** The patient population was subdivided between the different centers.

	Alessandria (N = 8)	Catania (N = 20)	Foggia (N = 22)	Messina (N = 15)	Palermo (N = 4)	Pisa (N = 17)	Trieste (N = 162)	Total (N = 248)	* p * -Value
** Age **									0.009 ^1^
Mean (SD)	60.8 (17.1)	60.9 (14.8)	59.5 (13.3)	53.0 (18.2)	70.5 (16.3)	69.5 (11.0)	65.5 (14.1)	64.0 (14.6)	
Range	36.0–78.0	29.0–82.0	32.0–80.0	18.0–83.0	54.0–85.0	41.0–87.0	18.0–89.0	18.0–89.0	
** Gender **									0.451 ^2^
Female	3.0 (37.5%)	6.0 (30.0%)	5.0 (22.7%)	8.0 (53.3%)	1.0 (25.0%)	7.0 (41.2%)	70.0 (43.2%)	100.0 (40.3%)	
Male	5.0 (62.5%)	14.0 (70.0%)	17.0 (77.3%)	7.0 (46.7%)	3.0 (75.0%)	10.0 (58.8%)	92.0 (56.8%)	148.0 (59.7%)	
** BMI **									0.458 ^1^
Mean (SD)	26.8 (4.0)	26.8 (6.2)	26.1 (5.5)	30.1 (6.8)	27.7 (1.0)	26.5 (5.4)	26.5 (5.9)	26.8 (5.8)	
Range	21.5–33.6	17.3–43.0	11.7–38.0	18.8–48.5	26.4–28.9	19.5–37.2	16.5–48.4	11.7–48.5	
** Admission **									0.058 ^2^
Medical	0.0 (0.0%)	11.0 (55.0%)	10.0 (45.5%)	3.0 (20.0%)	1.0 (25.0%)	4.0 (23.5%)	66.0 (40.7%)	95.0 (38.3%)	
Surgical	8.0 (100.0%)	9.0 (45.0%)	12.0 (54.5%)	12.0 (80.0%)	3.0 (75.0%)	13.0 (76.5%)	96.0 (59.3%)	153.0 (61.7%)	
**Site of Infection**									<0.001 ^2^
Other	1.0 (12.5%)	2.0 (10.0%)	8.0 (36.4%)	2.0 (13.3%)	0.0 (0.0%)	2.0 (11.8%)	28.0 (17.3%)	43.0 (17.3%)	
Abdomen	3.0 (37.5%)	7.0 (35.0%)	8.0 (36.4%)	12.0 (80.0%)	2.0 (50.0%)	10.0 (58.8%)	89.0 (54.9%)	131.0 (52.8%)	
Genitourinary	4.0 (50.0%)	0.0 (0.0%)	0.0 (0.0%)	0.0 (0.0%)	2.0 (50.0%)	2.0 (11.8%)	21.0 (13.0%)	29.0 (11.7%)	
Lung	0.0 (0.0%)	11.0 (55.0%)	6.0 (27.3%)	1.0 (6.7%)	0.0 (0.0%)	3.0 (17.6%)	24.0 (14.8%)	45.0 (18.1%)	
** SAPSII **									0.799 ^1^
Mean (SD)	51.5 (17.6)	47.0 (12.1)	49.1 (15.7)	54.1 (19.3)	53.0 (10.4)	47.6 (15.6)	51.4 (16.0)	50.8 (15.8)	
Range	35.0–87.0	28.0–68.0	22.0–89.0	26.0–95.0	44.0–63.0	18.0–77.0	7.0–97.0	7.0–97.0	

^1^ Linear model ANOVA; ^2^ Pearson’s Chi-squared test.

**Table 2:** List of rTO-PIRO criteria with *p*-values from the Kaplan–Meier analysis. Those significant (below 0.05) or almost significant included in the SORRISO score are in bold, for which Kaplan–Meier curves are available in the Appendix A.1.

Items	Criteria	*p*-Value
Predisposition	**Institute**: Trieste vs. others	0.31
	**Gender**: Male vs. female	0.72
	**Age**: Under 65 years old vs. over 65 years old	0.12
	**Body Mass Index**: Under 25 vs. over 25	0.94
	**P1_TOPIRO** (revised): Any cancer vs. no cancer	0.078
	**P2_TOPIRO**: Colonization by MDR bacteria and/or candida vs. no colonization	0.75
	**P3_TOPIRO** (revised): Neutropenia or immunosuppressive drugs (monoclonal/steroids/mycophenolate/cyclosporin) or allogenic stem cell transplant or splenectomy or AIDS vs. none	0.00042
Insult	**Type of admission**: Surgical vs. medical	0.016
	**Site of infection**: Abdomen vs. others	0.067
	Positive culture isolation: **Gram-positive bacteria** vs. none	0.52
	Positive culture isolation: **Gram-negative bacteria** vs. none	<0.0001
	Positive culture isolation: **Fungal** vs. none	0.18
	Positive culture isolation: **Others** vs. none	0.54
	Positive culture isolation: **Polymicrobial** vs. none	0.23
	**I1_TOPIRO**: Necrotizing fasciitis, invasive meningococcal/pneumococcal diseases, Streptococcus pyogenes; CA-MRSA vs. none	0.47
	**I2_TOPIRO**: MDR infections or nosocomial infections vs. none	0.0017
	**I3_TOPIRO**: Secondary/tertiary peritonitis vs. none	0.036
Response	**R2_TOPIRO**: IgM < 60 mg/dL vs. IgM ≥ 60 mg/dL (at day 0)	0.29
	**R3_TOPIRO**: PCT > 10 ng/L and CRP >20 mg/dL vs. none	0.98
	**R4_TOPIRO** (revised): PCT > 100 ng/L vs. none	0.29
	**R5_TOPIRO** (revised): Disseminated intravascular coagulation vs. none	<0.0001
	**SAPSII Score**: Under 50 vs. over 50	<0.0001
Organ	**Septic shock** vs. none	0.081
	**Lactate dosage** (at day 0): Lactate ≥ 2 mmol/L vs. lactate < 2 mmol/L	0.096
Therapy	**Timing of eIg** infusion (72 h): Early vs. late	0.04
	**Adequacy of antibiotic therapy**: Yes vs. no	0.021

**Table 3:** Calculation of the SORRISO Score.

Item	Criteria	Value
Predisposition	P1_TOPIRO no cancer	+1.5
	P3_TOPIRO no immunosuppression	+2.6
Insult	Type of admission: Surgical	+1.5
	Site of infection: Abdomen	+1.3
	Isolation: Gram-negative bacteria	+2.2
	I2_TOPIRO: MDR infections or nosocomial infections	+1.8
	I3_TOPIRO: Secondary/tertiary peritonitis	+1.2
Response	R5_TOPIRO (revised): No SIC	+2.8
Therapy	Timing of eIg: ≤72 h	+2.3
	Adequacy of antibiotic therapy: Yes	+3.1

Note. Estimates represent the log odds of “OUTCOME = dead” vs. “OUTCOME = alive”.

Note. The cutoff value is set to 0.5.

Note. The cutoff value is set to 0.5.

**Figure A1:**
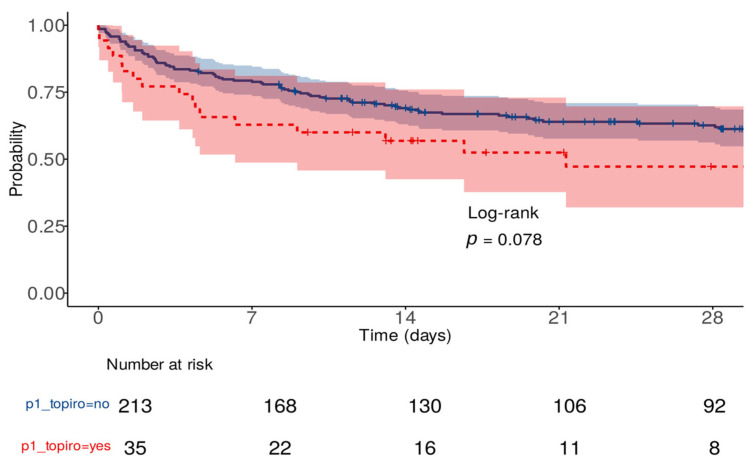
Kaplan–Meier curve for P1_TOPIRO (revised): any cancer vs. no cancer.

**Figure A2:**
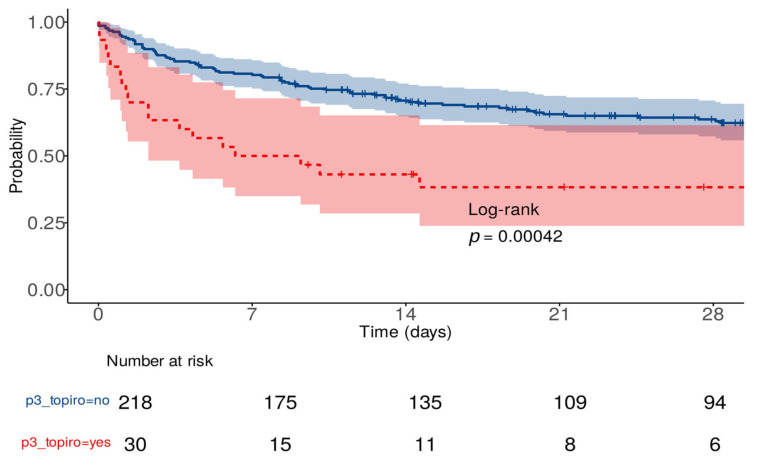
Kaplan–Meier curve for P3_TOPIRO (revised): Neutropenia or immunosuppressive drugs (monoclonal/steroids/mycophenolate/cyclosporin) or allogenic stem cell transplant or splenectomy or AIDS vs. none.

**Figure A3:**
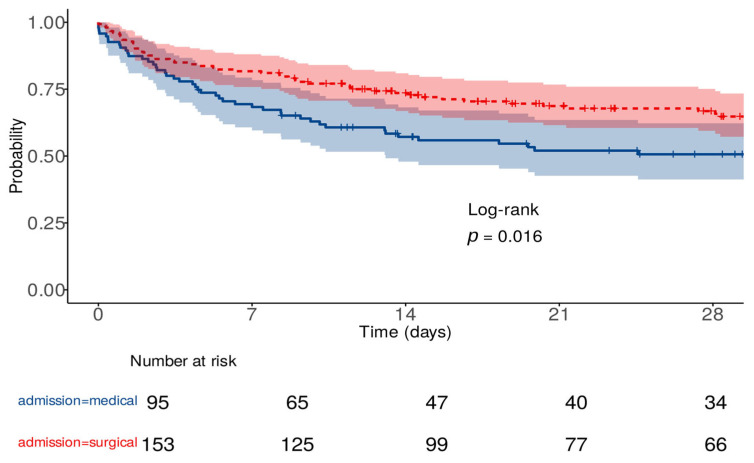
Kaplan–Meier curve for type of admission: surgical vs. medical.

**Figure A4:**
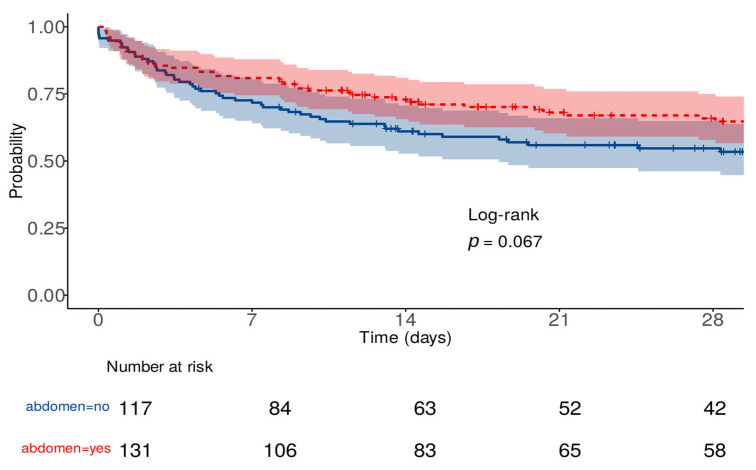
Kaplan–Meier curve for site of infection: abdomen vs. others.

**Figure A5:**
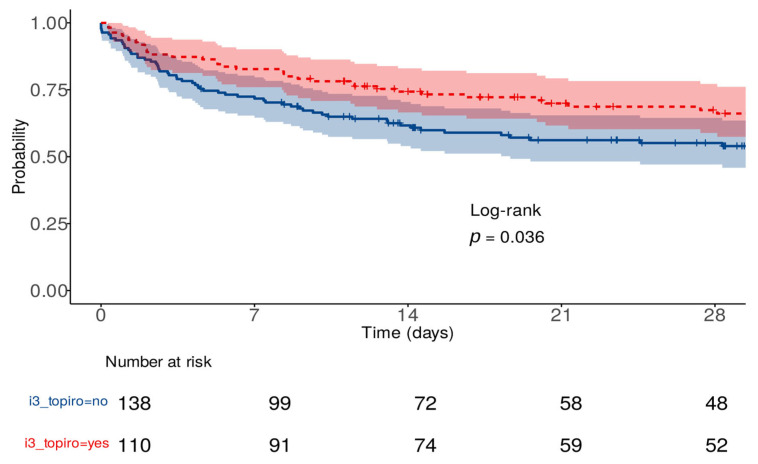
Kaplan–Meier curve for I3_TOPIRO: Secondary/tertiary peritonitis vs. none.

**Figure A6:**
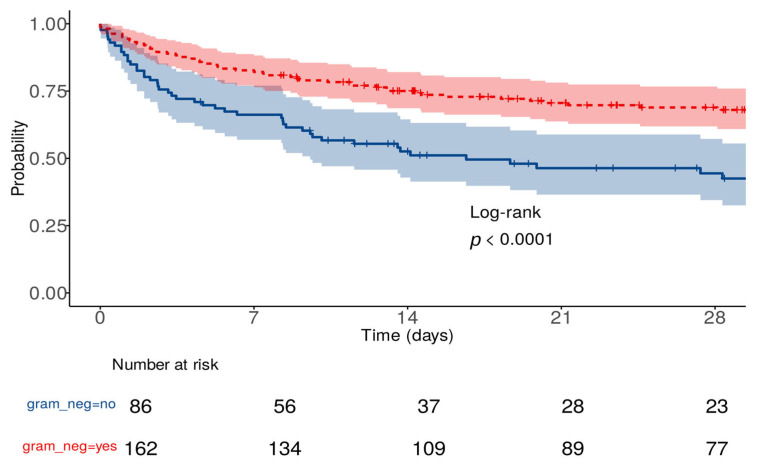
Kaplan–Meier curve for positive culture isolation: Gram-negative bacteria vs. none.

**Figure A7:**
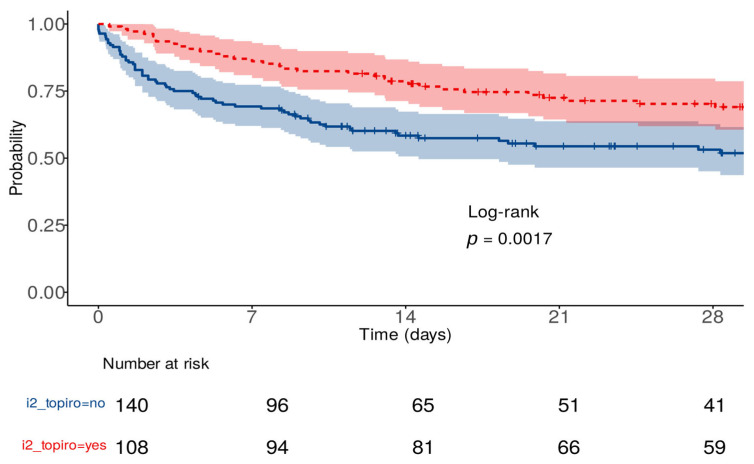
Kaplan–Meier curve for I2_TOPIRO: MDR infections or nosocomial infections.

**Figure A8:**
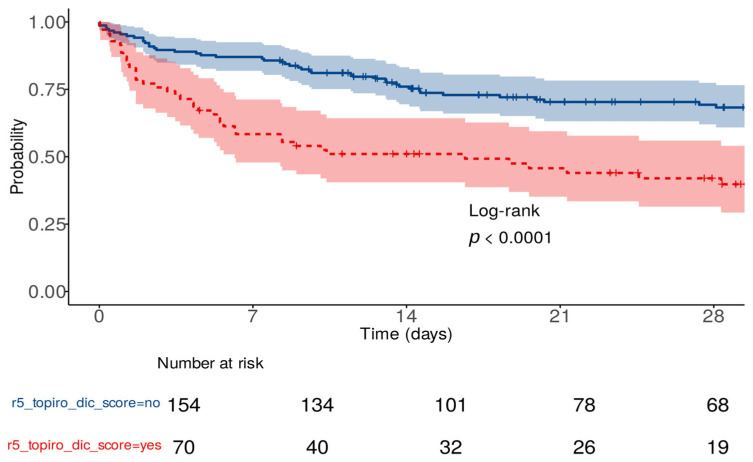
Kaplan–Meier curve for R5_TOPIRO (revised): Disseminated intravascular coagulation vs. none.

**Figure A9:**
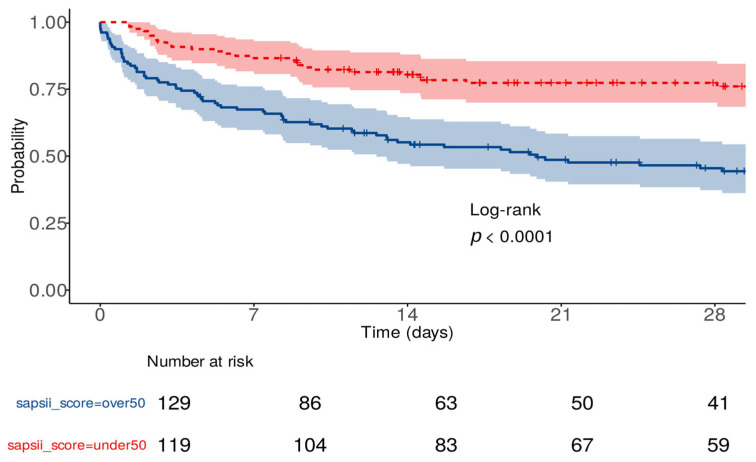
Kaplan–Meier curve for SAPSII Score: under 50 vs. over 50.

**Figure A10:**
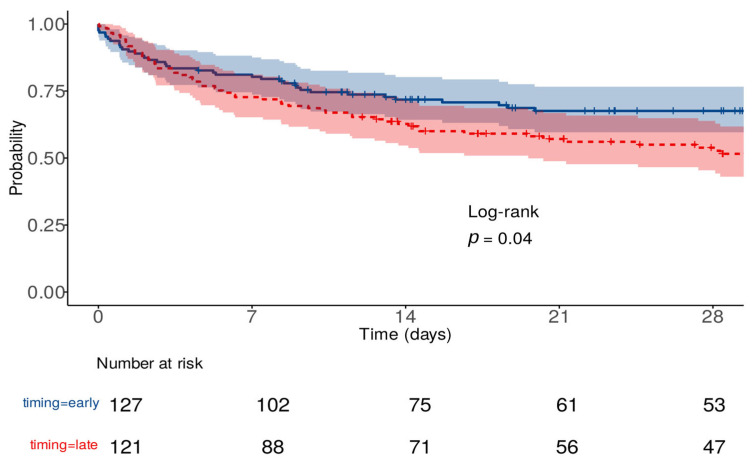
Kaplan–Meier curve for timing of eIg infusion (72 h): early vs. late.

**Figure A11:**
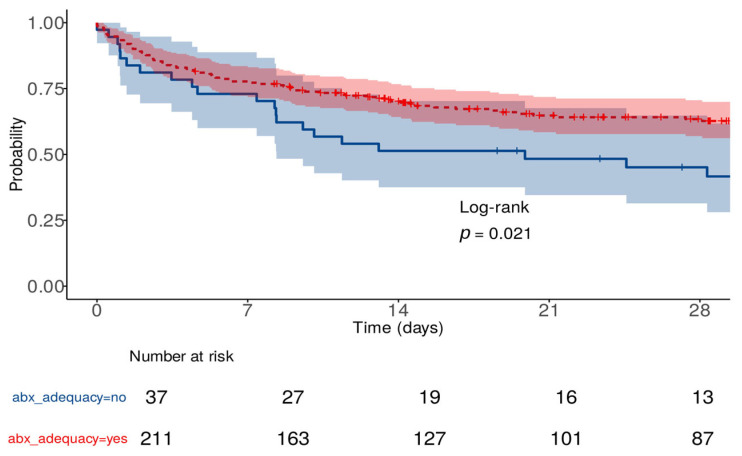
Kaplan–Meier curve for adequacy of antibiotic therapy: yes vs. no.

## Data Availability

The datasets presented in this article are not readily available due to GDPR restrictions.

## Abbreviations

The following abbreviations are used in this manuscript:

eIg	Intravenous Immunoglobulins Enriched with IgA and IgM
SORRISO	Studio Osservazionale Registro Rianimazioni Italiane Sepsi Ospedaliere
ICU	Intensive Care Unit
AUC	Area Under the Curve
SCC	Surviving Sepsis Campaign
IvIgs	Intravenous Immunoglobulins
EBM	Evidence-Based Medicine
RCTs	Randomized Clinical Trials
ROEs	Rules Of Engagement
SIC	Sepsis-Induced Coagulopathy
MDR	Multidrug Resistance
BP	Blood Purification
ES	Early Starters
LS	Late starters
S	Survivors
NS	Non-Survivors
PIRO	Predisposition, Insult, Response, Organ failure
MA	Meta-Analysis

## Appendix A

### Appendix A.2. Binomial Logistic Regression for the Calculation of the SORRISO Score

**Table:**

Model Fit Measures				
Model	Deviance	AIC	BIC	R^2^_McF_
1	289	293	300	0.126

**Table:**

Model Coefficients—OUTCOME						
		95% Confidence Interval				
Predictor	Estimate	Lower	Upper	SE	Z	*p*
Intercept	2.779	1.649	3.909	0.5767	4.82	<0.001
SCORE_tot	−0.246	−0.329	−0.162	0.0425	−5.78	<0.001

Note. Estimates represent the log odds of “OUTCOME = dead” vs. “OUTCOME = alive”.

#### Appendix A.2.1. Prediction Cutoff Plot

**Table:**

Classification Table			
	Predicted		
Observed	Alive	Dead	% Correct
alive	128	25	83.7
dead	54	41	43.2

Note. The cutoff value is set to 0.5.

**Table:**

Predictive Measures			
Accuracy	Specificity	Sensitivity	AUC
0.681	0.837	0.432	0.731

Note. The cutoff value is set to 0.5.

### Appendix A.3. Cox Regression Summary and Table for the Calculation of the SORRISO Score Cutoff

When SCORE_tot increases 1 unit, the hazard increases 0.85 (0.81–0.89, *p* < 0.001) times.

**Table:**

Explanatory	Levels	All	HR (Univariable)	HR (Multivariable)
	Mean (SD)	13.5 (3.8)	0.85 (0.81–0.89, *p* < 0.001)	0.85 (0.81–0.89, *p* < 0.001)

**Model Metrics:** Number of data frames = 248, number of models = 248, missing = 0, number of events = 110, concordance = 0.696 (SE = 0.025), R-squared = 0.165 (max possible = 0.988), likelihood ratio test = 44.842 (df = 1, *p* = 0.000).

**Table:**

Cutoff Point	Statistic
13.7	6.33
